# Two-dose varicella vaccine effectiveness in China: a meta-analysis and evidence quality assessment

**DOI:** 10.1186/s12879-021-06217-1

**Published:** 2021-06-09

**Authors:** Zhujiazi Zhang, Luodan Suo, Jingbin Pan, Dan Zhao, Li Lu

**Affiliations:** Department of Immunization and Prevention, Beijing Center for Disease Prevention and Control, Beijing Research Center for Preventive Medicine, He Ping Li Zhong Jie No.16, Dongcheng District, Beijing, 100013 China

**Keywords:** Varicella vaccine, Vaccine effectiveness, Meta-analysis, GRADE

## Abstract

**Background:**

The objectives of this review were to evaluate the vaccine effectiveness (VE) of the two-dose varicella vaccine for healthy children in China and explore the application of the approach of Grades of Recommendation, Assessment, Development, and Evaluation (GRADE) in observational studies on VE.

**Methods:**

We searched for observational studies on two-dose varicella VE for children in China aged 1–12 years that were published from 1997 to 2019, and assessed the quality of each study using the Newcastle Ottawa Scale (NOS). We used meta-analysis models to obtain the pooled two-dose VE, and the studies were divided into subgroups and analysed according to whether or not it was an outbreak investigation and its NOS score. The quality of evidence of VEs were rated by approach of the GRADE system.

**Results:**

A total of 12 studies and 87,196 individuals were included. The pooled two-dose VE was 90% (95% confidence interval [CI]: 69–97%). The VE of outbreak studies (87% [95% CI: 76–93%]) was lower than non-outbreak studies (99% [95% CI: 98–99%]). There was no significant difference in VEs by different NOS quality. The quality of the evidence assessment of pooled two-dose VE was “low”, which was rated down by one category in limitations and publication bias respectively and rated up by two category in large effect. The quality of evidence assessment in subgroup of NOS score ≥ 7 was “moderate”.

**Conclusions:**

The VE of two-dose varicella vaccine is relatively high in preventing varicella, and is recommended for countries which need further control for varicella. However, higher quality evidence is needed as a supplement for stronger recommendations. The approach of GRADE could be applied for rating the quality of evidence in observational study.

**Supplementary Information:**

The online version contains supplementary material available at 10.1186/s12879-021-06217-1.

## Background

Varicella is a highly contagious disease caused by the Varicella-zoster virus (VZV), which is associated with fever and a generalized pruritic vesicular rash [1]. As varicella vaccines are available globally and introduced into the immunization program for children in some countries, morbidity and mortality associated with the disease has been reduced successfully in recent years [2, 3]. The first varicella vaccine (Varilrix) was introduced in China by GlaxoSmithKline Biologicals (Rixensart, Belgium) in 1997. However, it was not until the introduction of domestic vaccines in 2000 that varicella vaccines were widely used in China [4]. Most children were given one-dose schedule according to the instructions from the manufacturer. Although we succeeded in reducing varicella-related morbidity and mortality in the following years, it soon reached a stable level instead of continuously decreasing [5]. Varicella outbreaks still occurred frequently, especially in schools and kindergartens with a coverage rate of nearly 100% [6–8].

Since 2012, two-dose schedule started to recommend in some districts of China for further control of the outbreaks and the prevalent of varicella [9]. As a result, the coverage rate of the varicella vaccine has generally improved, with a one-dose coverage rate of 80–93% and a two-dose coverage rate of 48.7–72.9% [10–12]. The schedule transformation also provided a field to access the vaccine effectiveness (VE) of the two-dose vaccine, some studies confirming that the VE of the two-dose vaccine is indeed significant different from that of the one-dose vaccine. However, the sample sizes of those studies were usually insufficient and the outcomes were not always the same [6, 13, 14]. Therefore, we conducted a systematic literature review and meta-analysis to assess a more authentic effectiveness of the two-dose vaccine and provide more evidence for adjusting varicella immunization strategies at the national level.

The approach of Grades of Recommendation, Assessment, Development, and Evaluation (GRADE) was proposed to rate the quality of evidence and grading strength of recommendations by the GRADE Working Group in 2004 [15]. This has been adopted by 28 international organizations, such as the World Health Organization and the Cochrane Collaboration. the framework offers a transparent and structured process for developing and presenting evidence summaries for systematic reviews and guidelines in health care and carrying out the steps involved in developing recommendations [16]. However, at the time of writing, experience with GRADE has only been used for the evaluation of therapeutic interventions research and clinical questions rather than for public health and health systems questions [17, 18]; thus, there was limited experience for reference. In this study, the approach of GRADE was applied to assess the quality of evidence provided by observational studies on VE evaluation in order to provide a new train of thought and reference for other researchers.

## Methods

### Inclusion criteria

The Inclusion criteria were as follows: 1) observational study on two-dose varicella VE; 2) the study population was healthy Chinese children aged 1–12 years old; 3) the intervention was immunization with two doses of the varicella vaccine; 4) the comparison was no immunization with the varicella vaccine; and 5) the outcome was VE in the studied population. Studies on clinical trials, methodology, molecular biology, vaccine development, animal studies, popular science lectures, newspaper articles, and literature reviews were excluded. For articles that were published repeatedly, we selected the one with most complete information.

### Search strategy

We searched for articles published from 1997, when the first varicella vaccine was introduced into China, to September 2019, in the following databases: China National Knowledge Internet, Wan Fang Database, Chinese Biomedical Literature Service System (SinoMed), PubMed, EMBASE, and Cochrane Library. We used search terms including “varicella”, “chickenpox”, “vaccine”, “effective”, “effectiveness”, and “protective”. “Chinese” or “China” were used when searching for English articles to identify articles that presented data on varicella VE in the Chinese population. Reference lists of selected articles and key published reviews [19–21] were also hand-searched (Supplementary file 1). This systematic review and meta-analysis was performed according to the Preferred Reporting Items for Systematic Reviews and Meta-Analyses (PRISMA) protocol [22].

### Study selection

We used NoteExpress (3.2.0.7629) to eliminate duplicates. Two reviewers (ZZ and LS) screened the studies based on the inclusion criteria described above independently. Disagreements were resolved by consensus or consultation with a third member of the team (LL).

### Data extraction

Data extraction forms were developed for the methodological quality assessment of individual studies, sub-analysis, and evidence quality rating. Two reviewers (ZZ and LS) performed the data extraction independently. Authors of original articles were contacted in the event of missing or inaccurate information. The VE was calculated by the reviewers when it was not reported but there were enough data to estimate.

For each included study in analysis, we abstracted information on the characteristics of the study (authors, study year, study design and analysis, cases and information sources, and conflicts of interest), population, intervention (vaccination status, vaccine type, age at vaccination, number of doses, and interval between doses), and outcome (case definition, diagnosis and reporting, observation duration, and loss to follow-up). VE was calculated by comparing varicella attack rates among vaccinated and non-vaccinated populations or by comparing the vaccination status of cases and non-cases during varicella outbreaks [23, 24].

### Methodological quality assessment

Two reviewers (ZZ and LS) independently evaluated the methodological quality for included studies, and any disagreements were resolved by consensus or by consulting a third member of the team (LL). The Newcastle-Ottawa Scale (NOS) [25, 26] was adapted to evaluate the selection, comparability, and outcome/exposure of the study, using eight items for case-control studies and cohort studies. A maximum of nine points was assigned to each study. Studies with a score of 1–3, 4–6, or 7–9 was considered as being of “low”, “intermediate”, or “high” methodological quality, respectively.

### Meta-analysis

We used Stata (version 15.0) to perform all statistical calculations in this meta-analysis. Data were combined and estimated for a pooled relative risk (RR) or odds ratio (OR), depending on the study design, and 95% confidence intervals (CIs) were calculated. The RRs/ORs and CIs of the subgroups were analysed by a outbreak or non-outbreak investigation and NOS score. VE was calculated as (1 − RR/OR) × 100%. The *I*^2^ value was used to assess heterogeneity [27]. We considered *I*^2^ ≥ 50 as being of high heterogeneity and chose random effects models, and *I*^2^ < 50 as being of low heterogeneity and chose fixed effects models. Publication bias was assessed using Egger’s regression test; *P* < 0.05 indicates evidence of publication bias.

### Evidence quality rating

We used GRADEpro (version 3.5) to rate the quality of evidence of VEs by means of the GRADE system; the ratings were completed by two reviewers (ZZ and LS) and any disagreements were resolved by consulting a third member of the team (LL). The quality of evidence was divided into four categories: “high”, “moderate”, “low”, and “very low”. This was to reflect our confidence that the estimated of the VEs were correct. Randomized trials were considered high quality evidence, and observational studies as low quality. Five factors can decrease the quality of evidence: limitations, inconsistency, indirectness, imprecision, and publication bias. Three factors can increase the quality of evidence: large effect, plausible residual confounding, and dose-response gradi [28].

## Results

### Search results

**Fig. 1:**
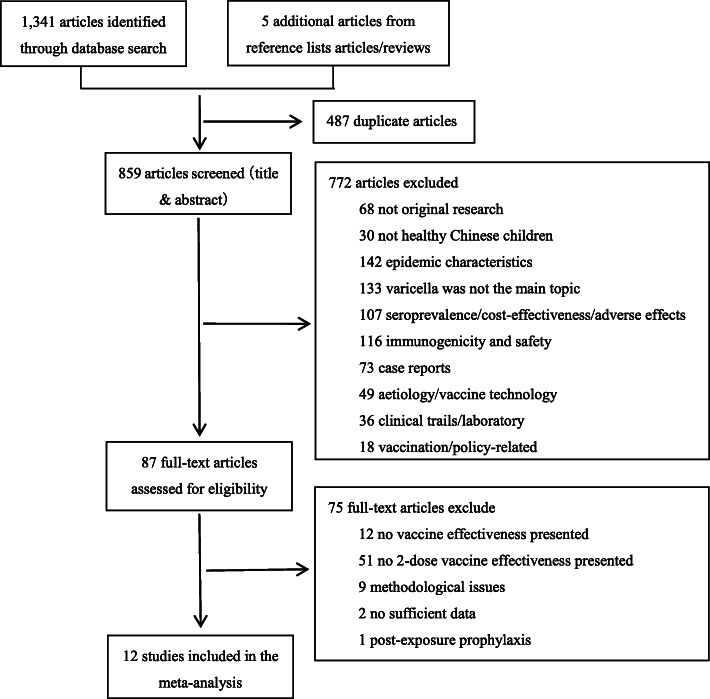
Study selection: Two reviewers selected the studies independently according to the Preferred Reporting Items for Systematic reviews and Meta-Analyses (PRISMA). Any disagreements were resolved by consulting the third person

We identified a total of 1341 articles from the literature search and five extra articles; 487 duplicate articles were excluded. After reviewing titles and abstracts, 772 articles were found to not be original research, or were on other topics or had different populations. Of the remaining 87 articles, we read the full-text and found that most were not on two-dose VE or even general VE. We also excluded articles that did not provide correct and sufficient data. Finally, 12 original studies met our inclusion criteria for meta-analysis (Fig. 1; references listed in Table 1) [6, 13, 14, 29–37].

**Table 1:** Basic information of studies included in this meta-analysis

Author	Study Year	Study Design	Study Setting, Range of Age/Average Age	Participants	Outbreak or Not	Selection^b^	Comparability^b^	Outcome^b^	Total Score^b^
Li Lu	2012	Retrospective cohort	Elementary school, 5–8 y	8	Y	3	2	3	8
Pang Hong	2011–2012	Retrospective cohort	Elementary school, 6–12 y^a^	123	Y	3	1	3	7
Sui Haitian	2015	Retrospective cohort	Elementary school, 6–12 y^a^	234	Y	1	1	2	4
Zhu Qi	2014–2015	Retrospective cohort	Kindergarten and elementary school, 5–10 y^a^	325	Y	3	1	3	7
Wei Yujia	2015	Retrospective cohort	Elementary school, 8 y	65	Y	3	1	3	7
Xingqiang Pan	2009–2016	Retrospective cohort	Community, 3–8 y^a^	83,481	N	4	0	3	7
Sun Yuan	2016	Retrospective cohort	Elementary school, 7–12 y	817	Y	3	1	3	7
Wang Xu	2016	Retrospective cohort	Elementary school, 9–12 y	763	Y	3	1	3	7
Cai Jintang	2015–2016	Prospective cohort	Community, 2–6 y	79	N	3	2	1	6
Chen Jinsheng	2017	Retrospective cohort	Elementary school, 6–12 y	569	Y	3	1	3	7
Ni Zhaorong	2017–2018	Retrospective cohort	Elementary school, 6–12 y^a^	516	Y	2	1	2	5
Zhuang Lin	2018	Retrospective cohort	Elementary school, 6–11 y	216	Y	3	1	3	7

^a^Our estimate; age range not provided in the publication

^b^Selection (maximum:four stars); Comparability (maximum:two stars); Outcome (maximum:three stars); Total score (maximum:nine stars)

### Study characteristics

We included 12 studies on two-dose varicella VE which were all cohort studies. Of these, 10 studies were conducted during outbreak investigations and used a retrospective cohort study design, one used a retrospective cohort design based on data from the Information System of disease and immunization, and one used a prospective cohort based on community investigation. The population studied mainly included children in settings of elementary schools (10 out of 12) whose ages ranged from 5 to 12 years old; two additional studies were on local children from the community. A total of 87,196 individuals were included and the overall age range was from 2 to 12 years (Table 1).

### Methodological quality of study

According to the NOS, the overall median quality score was 6.6 (4–8). Nine studies (75%) scored above 7, which indicated high quality, while the other three studies (25%) scored 4–6, indicating intermediate quality (Table 1). The main reasons for a lower score were as follows: 10 studies had a poor representativeness of cohorts, two studies likely had selection bias of the individuals, 10 studies could not ensure the comparability of cohorts, three studies had no confirmed outcome records, and one study did not have a sufficient follow-up duration.

### Meta-analysis of 2-dose varicella vaccine effectiveness

**Fig. 2:**
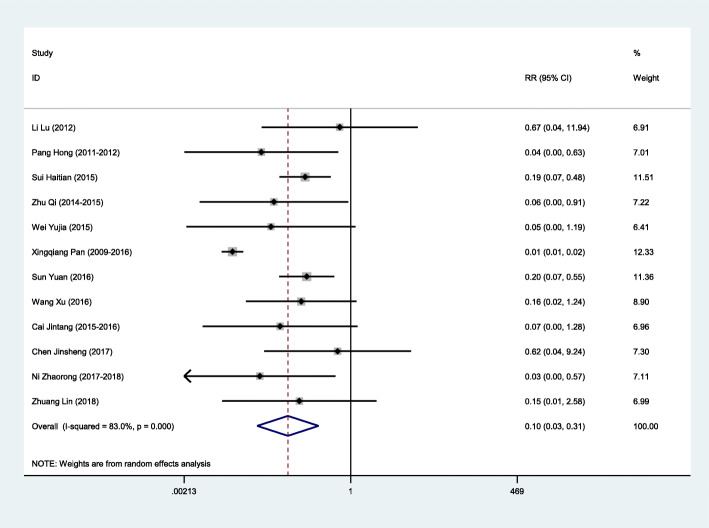
Random effects model of two-dose varicella VE for prevention varicella

**Fig. 3:**
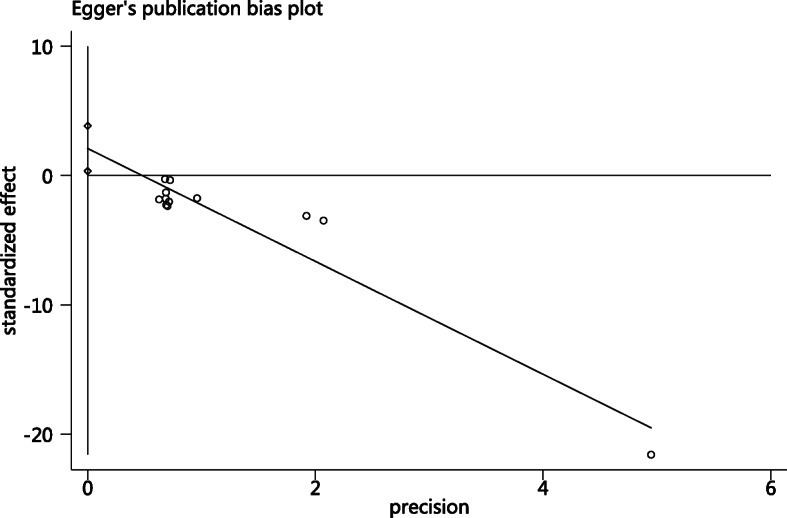
Funnel plot of two-dose varicella VE for prevention varicella

The meta-analysis of the 12 studies showed there was statistically significant heterogeneity (*I*^2^ = 83%); thus, pooled estimates of RR were calculated using a random effects model. The pooled two-dose VE for the prevention of varicella was 90% (95% CI: 69–97%; Fig. 2). There was evidence of publication bias indicated by Egger’s regression test (*p* = 0.024; Fig. 3). If one study with a large sample size (83,481) was removed [33], the pooled two-dose VE was 87% (95% CI: 77–93%, *I*^2^ = 0).

The studies were divided into subgroups and analysed according to whether or not it was an outbreak investigation and its NOS score. The heterogeneity of the studies with an NOS score of ≥7 was significant; therefore, we conducted a random effect model. The heterogeneity of other subgroups was less significant, and so fixed effect models were used. The polled two-dose VE of outbreak investigations was 87% (95% CI: 76–93%). This was lower than that of non-outbreak investigations, which was 99% (95% CI: 98–99%). Pooled VE estimates were similar in terms of the NOS score, which was 90% (95% CI: 60–97%) for scores ≥7 and 88% (95% CI: 71–95%) for scores < 7 (Table 2). Only one study [36] explained the vaccine type, a domestic vaccine produced by Beijing Tiantan biological products corporation limited, whose VE was 93% (95% CI across the invalid line).

**Table 2:** Results of two-dose varicella VEs analysis

Subgroup	No. of Study	Individuals of Study	*I*^*2*^ (%)	Polling Model	Publication Bias	RR (95%CI)	VE (%, 95%CI)
Overall	12	87,196	83	Random	0.024	0.10 (0.03 ~ 0.31)	90 (69 ~ 97)
Outbreak							
Y	10	3636	0	Fixed	0.334	0.13 (0.07 ~ 0.24)	87 (76 ~ 93)
N	2	83,560	34	Fixed	0.001	0.01 (0.01 ~ 0.02)	99 (98 ~ 99)
NOS score							
≥ 7	9	86,367	82	Random	0.020	0.10 (0.03 ~ 0.40)	90 (60 ~ 97)
< 7	3	829	0	Fixed	0.208	0.12 (0.05 ~ 0.29)	88 (71 ~ 95)

### The quality of evidence assessment in GRADE framework

The outcome of our evidence quality assessment was clinically diagnosed varicella, whose importance was rated crucial (8 points). Because all of the included studies were observational studies, the initial quality of the evidence was “low”. Overall, the evidence quality assessment of the polled two-dose VE was “low”. Limitations was rated down by one category as selection bias of the study populations was likely present. Inconsistency was not rated down as we were sure the reason of the significant heterogeneity was a study [33] that had a large sample size and higher RR. Indirectness was not rated down as the population, intervention, comparison and outcomes of the studies were consistent with what we studied in this meta-analysis. Imprecision was not rated down as the sample size of our study was relatively sufficient and the CI was moderate and did not cross the invalid line. Publication bias was rated down by one category as the Egger’s regression test indicated an evidence of publication bias. Large effect was rated up by two categories for a very large association of RR (< 0.2).

The quality of evidence assessment of the polled VEs of the subgroups showed that it was “moderate” in subgroups with an NOS score of ≥7 but “low” in other subgroups (Table 3).

**Table 3:** The summary of rating quality of evidence for two-dose varicella VEs

	Evidence Decrease					Evidence Increase			Quality
	limitations	Inconsistency	Indirectness	Imprecision	Publication Bias	large Effect	Plausible Confounding	Dose-response Gradi	
Pooled VE	serious^a^	no^b^	no	no	serious^c^	very large^d^	no	no	LOW
Outbreak									
Y	serious^a^	no	no	no	no	large^d^	no	no	LOW
N	serious^a^	no	no	no	serious^c^	very large^d^	no	no	LOW
NOS score									
≥ 7	no	no^b^	no	no	serious^c^	very large^d^	no	no	MODERATE
< 7	serious^a^	no	no	no	no	large^d^	no	no	LOW

^a^The results of NOS quality evaluation indicated that there was a high risk of bias

^b^We were sure the reason of the significant heterogeneity was a study had a large sample size and higher RR compared to the other studies

^c^Egger’s regression test indicated an evidence of publication bias

^d^We rated quality of evidence up by one category for RR associations less than 0.2, and up by two categories for associations less than 0.1

## Discussion

### Meta-analysis

This study systematically assessed the effectiveness of two-dose varicella vaccines used in China in a GRADE frame. In our analysis that included around 90,000 Chinese children from 12 studies, the two-dose varicella vaccine VE was 90%, similar to another global study (92%) [38]. Compared with the one-dose varicella vaccine VE (75%) [39] we studied previously, two doses of the vaccine could provide an extra 15% of protection than the one-dose. Compared with other vaccines, the two-dose varicella vaccine was shown to provide higher protection than the influenza vaccine (the VEs of different subtypes were approximately 33–73%) [40] and the two-dose rotavirus vaccine (63–72%) [41], and is similar with the measles vaccine (one dose was 90–95%) [42] and the measles-mumps-rubella vaccine (92% against measles [43] and 89% against rubella [44]). These means that the VE of the two-dose varicella vaccine is relatively high.

In China, only a few provinces have proposed a two-dose varicella vaccine recommendation as a non-expanded programme on immunization vaccine for Chinese children. Beijing was the first province that implemented a two-dose immunization strategy in 2012 [9], with the two-dose vaccine coverage reaching approximately 30–70% for different paediatric age groups in the following years [10]. At the same time, a significant decline of the varicella outbreak (52.7% [45]) was observed in the school setting, with the cases and the duration of the outbreaks decreasing as well. Thus, the two-dose varicella vaccine immunization strategy should be considered for children of the appropriate age in China as well as in other countries or areas that want improved control of varicella. Therefore, more studies and data are needed to confirm our results strongly.

The point estimate of the pooled VE of the outbreak was relatively lower than that of non-outbreak (87% versus 99%), which were similar to other outbreak and non-outbreak VE studies, respectively [46, 47]. There are no definitive explanations for this low effectiveness; it has been speculated that the force of infection may be high in some outbreaks or the degree of exposure may vary among study subjects. Decreased VE and failure of the vaccine to take effect could also be a reason for the outbreak-like breakthrough of varicella after the administration of a one-dose varicella vaccine [38]. Another reason may be that the studies with a large study population are more likely to attract researchers’ attention and be published [48].

### Evidence quality assessment

Through our assessment, the GRADE rating results of the evidence quality of two-dose varicella vaccine VE was “low”, which meant that our confidence in this VE estimate was limited and the true effectiveness may be substantially different from the estimated effectiveness. The main reasons of the quality decrease were as follows: 1) evidence from observational studies are generally initially “low”. All of the studies included in this meta-analysis were observational; most observational studies, even well-implemented ones, generally receive a quality evidence rating of “low” [49]; 2) there may be a high risk of bias. The NOS score range was 4–8, which indicated there were methodological defects in some studies; for instance, the individuals of most of the studies were recruited from an outbreak investigation, which was not representative enough and in which randomness existed. Furthermore, the balance between the two study groups was not fully compared, which may have led to a potential selection bias; 3) evidence of publication bias existed, suggesting that the higher RR may have come from few studies, which could have led to an overestimation of our pooled estimates. Thus, higher quality evidence in further studies is needed as a supplement to strengthen our confidence for a stronger recommendation.

We rated the evidence quality of NOS scores ≥7 as “moderate”, which meant we were moderately confident in the effect estimate and that the true effectiveness was likely to be close to the estimated effectiveness. It could be proven that the high-quality evidence or the strong recommendations need to be based on high-quality studies. Compared with the previous effectiveness estimate and quality assessment of the one-dose of varicella vaccine [39], which was studied relatively earlier, the studies included in this VE estimate were further improved in terms of information collection and presentation, case definition and exclusion, and paper writing.

However, we still found that the rating of the quality of some studies decreased due to information omission or inaccurate expression, rather than due to actual defects in methodology; this could likely explain why there was no significant difference in VE between studies in terms of NOS score-based quality. Therefore, it is suggested that technical guidelines must be formulated for observational studies in general, or even for observational studies on VE evaluation, including study design, data analysis, thesis writing, and other technical steps, to avoid the aforementioned problems for researchers and to improve the overall quality of evidence in China or on a global scale.

In addition to the aforementioned problems listed with the GRADE rating that affected the quality of evidence, our study still has several limitations. The relatively small number of study on two-dose varicella vaccine VE probably lead to the inaccurate evaluation of VE, as one study apparently had a large sample size and higher RR that might have increased the estimated value. The majority of the studies did not provide enough data to allow for a more detailed analysis of the vaccine types (domestic vaccine or imported vaccine), disease severity, and age of subjects, all of which may have a potential effect on VE or the waning immunity related to the occurrence of breakthrough varicella. The approach of GRADE to rating quality of evidence has a great requirement for knowledge of researchers in related fields, and it cannot eliminate the subjective judgment of researchers [16]. However, based on the systematic and transparent manner of the evaluation, we reduced subjective influence through parallel evaluation by multiple researchers as much as possible.

## Conclusion

Available data from China showed that the VE of the two-dose varicella vaccine is relatively high. To prevent varicella, it is recommended that the two-dose immunization strategy could be considered for countries that need to further control varicella, but higher-quality evidence is needed as a supplement for a stronger recommendation. The approach of GRADE could be applied to rating quality of evidence in observational studies of VE. It is necessary to formulate a technical guideline for observational studies to improve the quality of evidence.

## Supplementary Information

**Additional file 1: Supplementary Table 1.** Search strategies and Results of Literature Search by Database.

## Data Availability

Data sharing is not applicable to this article as no datasets were generated or analysed here. All of the articles included in this review are publicly available.

## Abbreviations

VE: Vaccine effectiveness
GRADE: Grades of Recommendation, Assessment, Development, and Evaluation
NOS: Newcastle Ottawa Scale
VZV: Varicella-zoster virus
RR: Relative risk
CI: Confidence interval
