# Supplementary material for: Two-dose varicella vaccine effectiveness in China: a meta-analysis and evidence quality assessment
Source: BMC Infect Dis. 2021 Jun 9;21:543. doi: 10.1186/s12879-021-06217-1 (PMC8188742; doi:10.1186/s12879-021-06217-1)
Supplement: Supplementary file 1 — Additional file 1: Supplementary Table 1. Search strategies and Results of Literature Search by Database. [file 12879_2021_6217_MOESM1_ESM.docx]

| Database | Search strategies | Results |
| --- | --- | --- |
| Chinese Biomedical Literature Service System (SinoMed) | #1 "varicella vaccine"[Extend] OR "varicella vaccine, Live"[All] OR "varicella vaccine"[All] OR ("varicella"[All] AND "live attenuated"[All] AND "vaccine"[All]) #2 "effectiveness"[All]OR"effective"[All]OR"efficacy"[All] OR "effect"[All] OR "efficiency"[All] OR "protective"[All] OR "performance"[All] OR "valid"[Extend]  #1 and #2 | 246 |
| China National Knowledge Internet (CNKI) | SU='varicella'*'vaccine' and SU='effectiveness' + 'effective' + 'efficacy' + 'effect' + 'efficiency' + 'protective' + 'performance' + 'valid' | 209 |
| Wan Fang Database | #1 topic：varicella AND vaccine  #2 topic：effectiveness OR effective OR efficacy OR effect OR efficiency OR protective OR performance OR valid  #1 and #2 | 704 |
| Pubmed | Search ((((MeSH Terms: validation studies as topic OR Publication Type: validation studies OR validation* OR efficac* OR effec*)) AND (MeSH Terms: chickenpox vaccine OR varicella vaccine))) AND (china OR Chinese) Sort by: Best Match | 54 |
| Embase | ('chickenpox vaccine'/exp OR varicella vaccine) and ('validation study'/exp OR validation* OR efficac* OR effec*) OR AND (china OR Chinese) | 111 |
| Cochrane Library | (Mesh:validation studies as topic[explode all tree] OR OR validation* OR efficac* OR effec*) AND (Mesh:chickenpox vaccine[explode all tree] OR varicella vaccine ) AND (china OR Chinese) | 17 |

**Supplementary Table 1. Search strategies and Results of Literature Search by Database**
